# Evaluation of Hepatic Mitochondria and Hematological Parameters in Zidovudine-Treated B6C3F_1_ Mice

**DOI:** 10.1155/2012/317695

**Published:** 2012-04-01

**Authors:** Varsha G. Desai, Taewon Lee, Carrie L. Moland, William S. Branham, Roberta A. Mittelstaedt, Sherry M. Lewis, Julian E. A. Leakey, James C. Fuscoe

**Affiliations:** ^1^Division of Systems Biology, Center for Functional Genomics, U.S. FDA/National Center for Toxicological Research, 3900 NCTR Road, Jefferson, AR 72079, USA; ^2^Department of Information and Mathematics, Korea University, Jochiwon, Chungnam 339-700, Republic of Korea; ^3^Division of Genetic and Molecular Toxicology, U.S. FDA/National Center for Toxicological Research, 3900 NCTR Road, Jefferson, AR 72079, USA; ^4^Office of Scientific Coordination, U.S. FDA/National Center for Toxicological Research, 3900 NCTR Road, Jefferson, AR 72079, USA

## Abstract

The effects of 12-week exposure to zidovudine (AZT) at 400, 500, and 600 mg/kg/d were examined on expression of 542 mitochondria-related genes and mitochondrial DNA (mtDNA) copy number in the liver of male and female B6C3F_1_ mice to understand mitochondrial role in sex-related differences in development of lactic acidosis. Plasma lactate levels and hematologic parameters were also examined. Results indicated increased red blood cell (RBC) count in vehicle-treated controls, whereas a dose-related decline in the RBC count was noted in AZT-treated mice compared to the basal levels before treatments began. These decreases were associated with significant dose-related increases in mean corpuscular volume and mean corpuscular hemoglobin levels. This effect was greater in AZT-treated females compared to males. In both sexes, 12-week AZT or vehicle exposure significantly reduced plasma lactate levels compared to the basal levels. Results also showed modest, but significant, changes in the expression of genes associated with apoptosis and lipid metabolism at 600 mg/kg/d AZT. Neither drug nor sex influenced hepatic mtDNA copy number. Altogether, 12-week AZT exposure as high as 600 mg/kg/d did not impair hepatic mitochondria or induce lactic acidosis in B6C3F_1_ mice. However, AZT-mediated hematologic toxicity appeared to be greater in females compared to males.

## 1. Introduction

The use of nucleoside reverse transcriptase inhibitors (NRTIs) in treatment of HIV-1 infections has been associated with a wide range of severe side effects, including lactic acidosis, hepatotoxicity, pancreatitis, lipodystrophy, neuropathy, and hematotoxicity [1]. In addition, sex-related differences in the frequency and severity of NRTI-related adverse effects have been reported. It has been shown that women are less likely than men to tolerate NRTI therapy [2] and also are at higher risk for developing NRTI-related toxicities [3]. Possible risk factors for sex-related differences in NRTI-induced toxicities include differences in body mass index, sex hormones, activities of kinases, drug-metabolizing enzymes, and the transport and excretion of the drug [4, 5]. Nonetheless, the molecular basis for an increased risk of developing NRTI-induced toxicity in HIV-1-infected women still remains unclear.

The majority of NRTI-related complications are believed to be due to altered mitochondrial function caused by phosphorylated metabolites of NRTIs that can inhibit DNA polymerase gamma and/or result in mtDNA chain termination [6, 7]. DNA polymerase gamma is the mitochondria-specific polymerase required for replication and repair of the mtDNA that encodes critical proteins for energy production by oxidative phosphorylation. Inhibition of DNA polymerase gamma and/or damage to mtDNA by NRTIs would alter energy production, consequently impairing cellular and mitochondrial function. Inhibition of DNA polymerase gamma by NRTIs leading to the depletion of mtDNA levels has been reported [8, 9]. Several studies, however, have also shown that NRTIs, such as zidovudine (AZT), can impair mitochondrial function independent of its effect on DNA polymerase gamma. This drug can affect other cellular targets including proteins involved in mitochondrial bioenergetics [10, 11], various metabolic pathways, and mitochondrial membrane transporters [12, 13].

One of the established measures of impaired mitochondrial energy production is higher blood lactate levels, which ultimately could lead to a life-threatening condition called lactic acidosis. Although rare, lactic acidosis is considered one of the most serious complications of NRTI therapy. It poses a major health concern in HIV-1-infected women, especially pregnant women, who are at greater risk of developing this adverse event compared to men [14, 15]. Thus, there is evidence that mitochondrial dysfunction is involved in NRTI-mediated side effects. However, the association between altered mitochondrial function and sex-based differences in NRTI-induced adverse events, in particular, lactic acidosis, has not been established.

 Bearing in mind that higher plasma lactate levels have been associated with mitochondrial dysfunction and that sex-related differences exist in the respiratory/oxidative potential of hepatic mitochondria [16], understanding mitochondrial activity during NRTI exposure will be critical to gain important insights into the role of mitochondria in sex-related differences in the development of hyperlactataemia/lactic acidosis. To address this, we examined the expression levels of 542 mitochondria-related genes and relative mtDNA copy number in the liver of male and female B6C3F_1_ mice treated with AZT, a principal antiretroviral drug used in combination regimens to treat HIV-1 infection. Plasma lactate levels were measured during AZT treatment to determine possible hyperlactataemia. Based on clinical studies that demonstrate AZT-related hematologic toxicity [17, 18], the RBC count, hemoglobin (Hb), mean corpuscular volume (MCV), and mean corpuscular hemoglobin (MCH) levels were also measured during AZT treatments in these mice.

## 2. Materials and Methods

### 2.1. Animal Husbandry

 Male and female B6C3F_1_ mice were obtained from the breeding colony at the National Center for Toxicological Research (NCTR). Mice were raised in a pathogen-free environment and treated according to Institutional Animal Care and Use Committee guidelines. Animals were housed two per polycarbonate cage with hardwood chip bedding and maintained at 23°C with a relative humidity of 50%. The animals were conditioned to a 12 hr light/12 hr dark cycle with lights on from 0600 to 1800 hr daily and were given autoclaved NIH-31 diet and filtered tap water *ad libitum*.

### 2.2. Animal Treatments

 Beginning at 8 weeks of age, mice were administered AZT (Cipla, Mumbai, India) by gavage at 0, 400, 500, and 600 mg/kg body weight/day for 12 weeks. McTween, which is a mixture of 0.2% methylcellulose and 0.1% polysorbate 80, was used as the vehicle. The AZT doses of 400, 500, and 600 mg/kg/d were selected based on a prior study that showed altered expression levels of hepatic mitochondria-related genes at 240 mg/kg AZT in C3B6F_1_ *trp*53 transgenic mice [13]. Also, the AZT doses of the current study were selected to severely impair mitochondrial function, but not high enough to be fatal to the animal. The human therapeutic dose for AZT is 10 mg/kg body weight/day [19]. On the basis of body surface area (mg/m^2^), the mice on the current study were exposed to 3.2, 4.0, and 4.8 times the clinical therapeutic dose, respectively. Control mice were administered McTween by gavage. Each group consisted of six animals. Animals were weighed and monitored daily for abnormal clinical signs.

### 2.3. Tissue Collection

 Blood was collected from mice before treatment began and also during the course of the treatment. Blood was collected from the saphenous vein of each animal and a portion of the blood was transferred into Microtainer tubes containing sodium fluoride and potassium oxalate (BD Biosciences, Franklin Lakes, NJ) for the measurements of lactate levels. The remaining blood was transferred into Microtainer tubes containing EDTA (BD Biosciences) for the measurements of hematologic parameters (RBC count, Hb, MCV, MCH). Blood samples collected for lactate measurements were immediately centrifuged at 1000 ×g for 10 minutes at room temperature to separate the plasma. Following 12-week treatment, animals were humanely euthanized by excessive inhalation of gaseous CO_2_ 24 hr after the last dose. To avoid possible adverse effects of CO_2_ asphyxiation on lactate levels, 30 minutes prior to euthanization, blood was collected from the saphenous vein for lactate measurements. Immediately following euthanasia, the median lobe of the liver was removed, minced in RNA *later* (Ambion, Austin, TX) and stored at −20°C until expression analysis of mitochondria-related genes. Another piece of the liver was immediately frozen in liquid nitrogen and stored at −80°C until measurements of mtDNA copy number.

### 2.4. Measurement of Expression Levels of Mitochondria-Related Genes Using the Mouse MitoChip

 Extraction of total RNA from liver tissues, reverse transcription to cDNA and labeling with fluorescent cyanine dyes, hybridization of labeled cDNAs on MitoChips, washing and scanning of hybridized arrays were as described previously [20]. In brief, total RNA was isolated from liver tissues using Qiagen RNeasy mini kit (Qiagen, Valencia, CA) followed by quantification of extracted RNA on a spectrophotometer at 260 nm and 280 nm. The quality of RNA samples was further determined using the RNA 6000 LabChip and Agilent 2100 Bioanalyzer (Agilent Technologies, Palo Alto, CA) and RNA samples with RNA integration number higher than 8.5 were used for microarray experiment. DNase-treated RNA samples were reverse transcribed using random hexamer primers (Invitrogen, Carlsbad, CA) and SuperScript II reverse transcriptase (Invitrogen) in the presence of reverse transcription labeling mixture containing dNTPs (Invitrogen) and aminoallyl-dUTP (aa-dUTP, Sigma, St. Louis, MO). Following purification, the aminoallyl-cDNA pellet was coupled with fluorescent cyanine dyes (Cy3 or Cy5, Amersham Pharmacia, Piscataway, NJ). Stratagene Universal Mouse Reference RNA (UMRR, Agilent Technologies, Cedar Creek, TX) was used as a reference RNA. Experimental samples and UMRR were labeled with Cy5 and Cy3, respectively. Each Cy5-labeled sample was cohybridized with Cy3-labeled UMRR on a MitoChip arrays and hybridized at 42°C for 18 hours in a water bath. This was followed by washing of arrays with sodium chloride sodium citrate buffer containing different concentrations of sodium dodecyl sulphate prewarmed at 30°C. MitoChip arrays were printed at the National Center for Toxicological Research. Each slide consisted of two microarrays. Following hybridization, arrays were scanned with a GenePix 4000B scanner (Axon Instruments, Union City, CA) at 10 *μ*m resolution.

The expression level of each gene for a sample was estimated by the average of the log_2_ ratio of the fluorescence of Cy5 (the sample) relative to the fluorescence of Cy3 (the reference) from the two arrays on the slide. Each array consisted of 9 housekeeping genes as positive controls and 9 Arabidopsis genes as negative controls. The expression level of control genes (housekeeping genes and Arabidopsis genes) for a sample was averaged and used as a covariate to normalize the expression data. A relative fold-change in the expression level of a gene was calculated as the ratio of the average expression of treated samples to untreated controls. Differences associated with treatment, sex, and their interactions were estimated under a fixed effect linear model with adjustment for batch effects and normalizing covariates using the Generalized Linear Model procedure of SAS 9.1.3. The results were further classified into different Gene Ontologies using the database from Mouse Genome Informatics (http://www.informatics.jax.org/). Overall treatment effects on the groups of genes (molecular function/biological process) were tested using a modified meta-analysis method for combining correlated *P* values [21].

### 2.5. Isolation of DNA and Measurement of Relative mtDNA Copy Number in the Liver

Isolation of DNA and measurement of relative mtDNA copy number in the liver were as described previously [22]. In brief, approximately 20 mg of liver tissue was homogenized in phosphate buffered saline (PBS) containing ΦX174 DNA (replicative form; NE Biolabs, Beverly, MA) as a reference DNA. To this, RNace-It (Agilent Technologies, Santa Clara, CA), and phosphorylase b from rabbit muscle (Sigma-Aldrich, St. Louis, MO) were added, followed by the addition of PBS without phage DNA to a final volume of 500 *μ*L before incubation at 37°C for 2 hours. This was followed by addition of 90 *μ*L of a digestion buffer containing 180 mM TRIS-HCl (pH 8.0), 60 mM sodium EDTA, 10% SDS, and 1.2 mg proteinase K (Qiagen, Valencia, CA) and tubes were incubated at 55°C overnight. This digest was further incubated at 95°C for 15 minutes, cooled to room temperature, and briefly centrifuged. Isolation of DNA from the digest was performed using TRI Reagent LS (Sigma-Aldrich) according to manufacturer's instructions. The resulting DNA pellets were dissolved in 75 *μ*L of 8 mM NaOH, and the quality and quantity of the DNA was assessed with an ND-1000 spectrophotometer (NanoDrop, Wilmington, DE).

 Relative mtDNA copy number was determined by a modification of the quantitative real-time PCR [TaqMan (Applied Biosystems), Foster City, CA)] assay method developed by Myers and colleagues [22]. Samples were analyzed on an ABI 7500 Sequence Detection System (Applied Biosystems) using TaqMan MGB probes designed for sequences specific to a region of the mouse mtDNA D-loop and the ΦX174 DNA reference. A calibrator sample containing equal ratios of D-loop and ΦX174 sequence was run on every plate used for quantitative-PCR analysis to allow all of the samples to be expressed as an n-fold difference relative to the calibrator using SDS software v1.1 (Applied Biosystems) and the comparative *C*_*t*_ method [23]. Two-way ANOVA was performed to determine AZT- and sex-related effects on relative mtDNA copy number.

### 2.6. Measurements of Plasma Lactate Level and Hematological Parameters

Lactate measurements were carried out using a lactate kit according to the manufacturer's instructions (Trinity Biotech, Ireland) on a COBAS Mira Plus automated spectrophotometer (Roche, Nutley, NJ). Statistical significance of dose-related changes in lactate levels for each sex was tested under a fixed effects linear model with a covariate of dose levels. Statistical significance of sex-related differences in lactate levels between 0- and 12-week was tested by *t*-tests, and *P*-values less than 0.05 were considered significant. During the course of the treatments (0–11-week), the RBC count, Hb content, MCV, and MCH were measured every week on a hematology analyzer, the PENTRA 60 C+ (Horiba ABX, France), using ABX reagents to determine anemia. Statistical significance of dose-related changes for each sex and sex-related differences in hematological parameters between 0- and 11-week was evaluated by *t*-tests, and *P* values less than 0.05 were considered significant. For the estimation of differences and the statistical analysis, a Generalized Linear Model of SAS 9.1.3 was used.

## 3. Results and Discussion

The present study evaluated liver mitochondria in AZT-treated B6C3F_1_ mice to understand the role of mitochondria in sex-related differences in the development of lactic acidosis. Both male and female B6C3F_1_ mice were treated with 400, 500, or 600 mg/kg/d AZT at doses predicted to severely alter mitochondrial function leading to hyperlactataemia/lactic acidosis. Despite the relatively high doses of AZT, there was no increase in plasma lactate levels in these mice. However, other classical parameters of clinical toxicity observed with AZT were altered and in a sex-related manner.

During the course of AZT treatments, hematological parameters were measured through 11 weeks of treatment to monitor possible anemia in these mice. Following 11-week exposure, significant changes in hematological parameters were found at all AZT exposures (Table 1). An AZT-related decline in the RBC count was observed in both sexes. The decreases in the RBC counts were 13%, 11%, and 8% in male AZT-treated mice at 400, 500, and 600 mg/kg/d, respectively, whereas the decreases were between 16–26% in female mice compared to 0-week (Table 1), and these declines were significant at 400 and 600 mg/kg/d AZT only in female mice. This may indicate a greater influence of AZT on the RBC count in female mice than males and this sex-related difference was significant at the 500 mg/kg/d dose. The occurrence of anemia characterized by reduced Hb levels has been reported in HIV-1 infected patients on NRTI therapies [24, 25]. In the present study, however, the Hb contents were not significantly altered despite the decline in RBC counts at all AZT doses (Table 1). On the other hand, small increases in RBC and Hb contents were observed in male and female mice following 11-week vehicle treatment compared to 0-week, and the increase was significant only in RBC counts in male mice (Table 1). At present, a likely mechanism underlying significant findings in vehicle-treated mice is unclear. More experiments need to be conducted to evaluate these changes.

In addition to the decline in RBC counts, AZT-treated mice showed significant dose-related increases in MCV and MCH levels (Table 1). Male mice treated with 400, 500, or 600 mg/kg/d AZT had a 22%, 22%, or 29% increase, respectively, in MCV at 11-week compared to 0-week. In AZT-treated female mice, the MCV levels were increased by 29%, 29%, and 36% following 11-week treatment with 400, 500, and 600 mg/kg/d AZT, respectively. In both AZT-treated male and female mice, these changes in MCV were associated with increases in MCH levels. While male mice had 15%, 17%, and 22% increases in MCH at 400, 500, and 600 mg/kg/d AZT, respectively, female mice showed 19%, 20%, and 27% increases at 400, 500, and 600 mg/kg/d AZT, respectively. Thus, as for the RBC counts, a greater AZT influence was seen in female mice, and this sex-related difference was significant at 400 and 500 mg/kg/d doses for both MCV and MCH levels but not at 600 mg/kg/d (Table 1). It is possible that beyond a certain threshold high concentration of AZT may overcome the genomic differences between sexes.

The MCV represents the average size of the RBC, and MCH indicates the amount of Hb in each RBC. Dose-related increases in MCV and MCH levels in AZT-treated mice might suggest a compensatory mechanism in response to a dose-related decline in the RBC count, which may further explain nonsignificant changes observed in Hb contents in these mice. Corroborating these findings are animal studies that reported hematologic toxicity as evidenced by the decreased erythrocyte count, higher MCV and MCH levels, and increased incidence of macrocytic anemia during AZT exposure [26–28]. The increased MCV indicates larger than normal RBCs, which are known as macrocytes. Although Hb levels were not altered in AZT-treated mice, higher MCV and MCH levels in response to decreased RBC count may indicate the initial phase of macrocytic anemia in these mice. Interestingly, a sex effect was seen in both MCV and MCH levels, where AZT-treated female mice had greater increases than their male counterparts and these increases were significant at 400 and 500 mg/kg/d doses. Altogether, these findings suggest the occurrence of hematotoxicity in AZT-treated B6C3F_1_ mice at all doses and this toxicity appeared to be greater in female mice than in males.

Although an increase in plasma lactate levels was anticipated upon AZT exposure, 12-week dosing with AZT significantly reduced plasma lactate levels in both male and female mice compared to the levels before treatment began (Table 2). The decreases in lactate levels showed no AZT dose response and were also found in animals treated with vehicle alone. The mechanism(s) underlying these decreases in lactate levels is unclear. Control and treated mice were given McTween, which is a commonly used emulsifying agent in drug treatments of laboratory animals in toxicology studies. However, changes in lactate levels in vehicle-treated mice similar to those seen in AZT-treated mice suggest a likely influence of McTween on lactate levels rather than the AZT. In future studies, the use of sterile water as the drug vehicle may help avoid this possible confounding effect.

In toxicological studies, increased lactate levels have been related to mitochondrial dysfunction in various drug-induced toxicities [29, 30]. In the present study, however, none of the AZT doses significantly altered the transcription levels of genes involved in mitochondrial energy production in the liver of B6C3F_1_ mice. In view of these findings, lack of increase in plasma lactate levels in AZT-treated mice is not surprising. A moderate, but significant effect of AZT was evident only at 600 mg/kg/d on the expression levels of genes associated with apoptosis and lipid metabolism (Table 3). Expression levels of proapoptotic genes (*Bax*, *Bbc3*, and *Bnip3*) were significantly increased in the liver. Bbc3 induces a conformational change in Bax, which is essential for apoptotic events mediated through the intrinsic pathway [31], whereas Bnip3 induces apoptosis by opening the mitochondrial permeability pore with consequential membrane depolarization [32]. Although immunohistochemical evaluation was not performed in the liver tissues to verify apoptotic changes, increased expression of proapoptotic genes may indicate a cellular response to AZT exposure, probably to remove damaged cells. Supporting this interpretation are *in vitro* and *in vivo* studies that reported increased apoptosis during exposures to antiretroviral drugs, including AZT [33, 34].

AZT-treated mice also exhibited differential expression of genes involved in lipid metabolism at 600 mg/kg/d dose. Increased transcriptional level of acyl-coenzyme A synthase (*Acsl3*) was associated with decreased expression levels of acyl-coenzyme A dehydrogenases for short-chain (*Acads*), short/branched chain (*Acadsb*), and long-chain fatty acids (*Acadl*). Acsl3 is required for the activation of long-chain fatty acids, whereas Acads, Acadsb, and Acadl are involved in the initial step of fatty acid **β**-oxidation. Decreases in the expression levels of acyl-coenzyme A dehydrogenases in AZT-treated mice were modest; however, in long-term therapies, such changes may, in part, contribute to fat accumulation in the liver. Hepatic steatosis, a condition characterized by excess fat accumulation within liver cells, has been indicated as one of the complications of antiretroviral therapy in HIV-1 infected patients [35]. Overall, the effect of AZT on hepatic mitochondria was minimal in B6C3F_1_ mice. This was further supported by nonsignificant changes in mtDNA copy number in the livers of AZT-treated mice (Table 4). Expression levels of mtDNA-encoded genes critical in energy production also remained unaltered by AZT treatment (Table 3). In addition, AZT-treated mice did not show sex-related differences in the expression levels of mitochondria-related genes or mtDNA copy number in the liver. Sex-based differences in the expression levels were evident only in vehicle-treated controls: female mice showing higher expression levels compared to males, and these genes were associated with complexes I, III, and V of oxidative phosphorylation and the Krebs cycle (data not shown).

In contrast to the paucity of gene expression changes found in this investigation, a previous study from our laboratory using a different mouse model showed that the expression of 164 genes was significantly altered by AZT exposure [13]. The reasons for this dramatic difference may be attributed to differences in genotype, age, and exposure to AZT. In the previous study, a p53 haplodeficient mouse strain was used. The single functional copy of the p53 gene in this strain may allow cells with damaged mitochondria to survive, and thus express altered mitochondria-encoding genes, by partially inhibiting apoptosis. The ages of the mice in the two studies, as well as the duration of exposure, were also different with young animals being used in the prior study (exposed perinatally from gestation day 12 to 18 followed by continuation of same treatment from postnatal day 1 through 28 days of age) and adult animals (exposed from 8 weeks of age to 20 weeks of age) used in the present study. Further studies will be required to fully understand the possible influence of genotype and age on gene expression changes induced by AZT.

In conclusion, the AZT doses used in the present study, although as high as 600 mg/kg/d, did not induce hyperlactataemia/lactic acidosis in B6C3F_1_ mice. This could be due to the absence of an AZT effect on hepatic mitochondria as evidenced by nonsignificant changes in the expression levels of mitochondria-related genes: in particular, genes associated with oxidative phosphorylation or the copy number of mtDNA. It is possible that higher doses of AZT and/or longer duration of AZT exposure may be necessary to significantly impair hepatic mitochondrial function in order to develop hyperlactataemia/lactic acidosis in B6C3F_1_ mice. Alternatively, use of a more sensitive mouse model, such as the p53 haplodeficient mouse, may accelerate mitochondrial dysfunction and will provide better insights into the role of mitochondria in sex-associated differences in AZT-related adverse events. In B6C3F_1_ mice, AZT effect on mitochondria was limited to modest changes in the expression levels of genes associated with apoptosis and lipid metabolism and occurred only at the highest dose. A striking finding, however, was a sex-related difference in hematologic toxicity in AZT-treated mice: female mice exhibiting a greater response than males.

## Figures and Tables

**Table 1:** Levels of hematological parameters in B6C3F_1_ mice.

AZT dose (mg/kg/d)	RBC count (10^6^/mm^3^)				Hb content (g/dL)				MCV (*μ*m^3^)				MCH (pg)			
	0-week		11-week		0-week		11-week		0-week		11-week		0-week		11-week	
	Male	Female	Male	Female	Male	Female	Male	Female	Male	Female	Male	Female	Male	Female	Male	Female
0	9.9 ± 0.3	10.1 ± 0.5	11.0^a^ ± 0.1	11.5 ± 0.3	16.5 ± 0.6	16.9 ± 0.7	16.9 ± 0.5	18.2 ± 0.7	46.6 ± 0.2	47.0 ± 0.3	47.0 ± 2.0	48.5 ± 1.2	16.4 ± 0.3	16.6 ± 0.2	15.4 ± 0.5	15.8^a^ ± 0.3

400	8.7 ± 0.7	9.8 ± 0.5	7.6 ± 0.4	7.3^a^ ± 0.3	14.2 ± 1.2	16.5 ± 0.9	14.4 ± 0.9	14.4 ± 0.8	47.2 ± 0.3	46.8 ± 0.3	57.8^a^ ± 0.3	60.5^a,b^ ± 0.5	16.5 ± 0.1	16.7 ± 0.2	19.0^a^ ± 0.1	19.8^a,b^ ± 0.2

500	8.6 ± 0.7	10.0 ± 0.7	7.7 ± 0.1	8.4^b^ ± 0.1	14.1 ± 1.2	16.5 ± 1.2	14.8 ± 0.3	16.5^b^ ± 0.2	47.8 ± 0.3	46.7 ± 0.2	58.5^a^ ± 0.5	60.3^a,b^ ± 0.3	16.4 ± 0.1	16.4 ± 0.2	19.2^a^ ± 0.1	19.7^a,b^ ± 0.1

600	8.6 ± 0.7	8.8 ± 0.3	7.9 ± 0.5	6.7^a^ ± 1.1	14.1 ± 1.2	14.4 ± 0.4	15.6 ± 0.8	13.6 ± 2.1	46.6 ± 0.7	46.2 ± 0.6	60.3^a^ ± 0.7	63.0^a^ ± 1.0	16.2 ± 0.2	16.2 ± 0.1	19.7^a^ ± 0.2	20.6^a^ ± 0.4

^a^indicates a significant (*P* < 0.05) difference in hematological parameter at 11-week compared to 0-week for each sex.

^b^indicates a significant (*P* < 0.05) sex-based difference in hematological parameter in female mice compared to male counterparts at 11-week. Values are presented as the mean ± SEM (*N* = 4–6). Also, a significant (*P* < 0.05) dose-related response was noted in the RBC count, Hb content, MCV, and MCH level in both sexes.

**Table 2:** Plasma lactate levels in B6C3F_1_ mice.

AZT dose (mg/kg/d)	Plasma lactate level (mg/dL)			
	0-week		12-week	
	Male	Female	Male	Female
0	101.4 ± 10.3	126.5 ± 15.4	71.5 ± 1.8^a^	62.0 ± 3.2^a,b^
400	100.0 ± 3.7	92.6 ± 9.2	79.3 ± 8.0^a^	56.0 ± 5.8^a,b^
500	96.7 ± 3.2	87.5 ± 2.2	57.9 ± 5.2^a^	56.3 ± 4.2^a^
600	92.6 ± 4.2	91.2 ± 2.6	65.7 ± 4.3^a^	52.7 ± 2.3^a,b^

^a^indicates a significant (*P* < 0.05) difference in plasma lactate levels at 12-week compared to 0-week for each sex.

^b^indicates a significant (*P* < 0.05) sex-related difference in plasma lactate levels in female mice compared to male counterparts at 12-week.

Values are presented as the mean ± SEM (*N* = 4).

**Table 3:** Effect of AZT (600 mg/kg/d) on the expression levels of genes in the liver of B6C3F_1_ mice.

Gene description (gene symbol)	GenBank accession ID	FC	*P*
Apoptosis (18), *P* = 0.039			

Bcl2-associated X protein (*Bax*)	NM_007527	1.143	0.020
Bcl2 binding component 3 (*Bbc3*)	NM_133234	1.065	0.032
Bcl2/adenovirus E1B 19 k Da-interacting protein 1, NIP3 (*Bnip3*)	NM_009760	1.159	0.041

Lipid metabolism (23), *P* = 0.046			

Acyl-Coenzyme A synthetase long-chain family member 3 (*Acsl3*)	AK012088	1.166	0.001
Acyl-Coenzyme A dehydrogenase, long-chain (*Acadl*)	NM_007381	−1.159	0.025
Acyl-Coenzyme A dehydrogenase, short-chain (*Acads*)	NM_007383	−1.142	0.022
Acyl-Coenzyme A dehydrogenase, short/branched chain (*Acadsb*)	NM_025826	−1.225	0.011

Mitochondrial genes (13), *P* = NS			

NADH ubiquinone dehydrogenase, subunit 1 (*mt-Nd1*)	AK018753	1.080	0.310
NADH ubiquinone dehydrogenase, subunit 2 (*mt-Nd2*)	AU018363	1.080	0.280
NADH ubiquinone dehydrogenase, subunit 3 (*mt-Nd3*)	J01420	1.021	0.724
NADH ubiquinone dehydrogenase, subunit 4 (*mt-Nd4*)	AB042809	1.018	0.827
NADH ubiquinone dehydrogenase, subunit 4L (*mt-Nd4l*)	AA109866	1.048	0.526
NADH ubiquinone dehydrogenase, subunit 5 (*mt-Nd5*)	AU018713	1.050	0.564
NADH ubiquinone dehydrogenase, subunit 6 (*mt-Nd6*)	AA435281	1.081	0.144
Cytochrome b (*mt-Cytb*)	AU018811	1.013	0.861
Cytochrome c oxidase, subunit I (*mt-Co1*)	AU018394	1.014	0.829
Cytochrome c oxidase, subunit II (*mt-Co2) *	AU019143	1.054	0.449
Cytochrome c oxidase, subunit III (*mt-Co3*)	AB042432	1.018	0.765
ATP synthase, subunit 6 (*mt-ATP6*)	AF093677	1.008	0.906
ATP synthase, subunit 8 (*mt-ATP8*)	J01420	1.067	0.464

The bold numbers in parentheses indicate number of genes evaluated for the molecular function. *P *values in bold represent effect of AZT on the molecular function, which is a combined effect of AZT-related changes in expression levels of number of genes evaluated for the molecular function. FC indicates a relative fold change in mice treated with 600 mg/kg/d AZT compared to vehicle-treated control mice. Negative values of fold changes indicate down-regulation of genes. Mitochondrial genes important in energy production, for which 600 mg/kg/d AZT did not alter transcription, are also presented in the table. Sex-related differences in the expression levels of mitochondria-related genes were not observed in liver of AZT-treated mice. NS: Not significant.

**Table 4:** Relative mitochondrial DNA (mtDNA) copy number in the livers of B6C3F_1_ mice.

AZT dose (mg/kg/d)	B6C3F_1_ mice	
	Male	Female
0	12.02 ± 4.87 (*N* = 6)	8.46 ± 1.51 (*N* = 6)
400	8.46 ± 3.83 (*N* = 5)	7.37 ± 1.57 (*N* = 6)
500	18.69 ± 8.26 (*N* = 6)	8.95 ± 1.29 (*N* = 6)
600	11.66 ± 7.05 (*N* = 5)	10.08 ± 1.75 (*N* = 5)

The mtDNA copy number was based on quantitative real-time PCR measurements of D-loop concentration relative to the concentration of ΦX174RF added at a fixed concentration before DNA extraction. The numbers in parentheses indicate the number of animals in the group. Values are presented as the mean ± SEM (Standard error of the mean).
